# The Consistency of Beneficial Fitness Effects of Mutations across Diverse Genetic Backgrounds

**DOI:** 10.1371/journal.pone.0043864

**Published:** 2012-08-24

**Authors:** Victoria M. Pearson, Craig R. Miller, Darin R. Rokyta

**Affiliations:** 1 Department of Biological Science, Florida State University, Tallahassee, Florida, United States of America; 2 Department of Biological Sciences and Department of Mathematics, University of Idaho, Moscow, Idaho, United States of America; University of Ottawa, Canada

## Abstract

Parallel and convergent evolution have been remarkably common observations in molecular adaptation but primarily in the context of the same genotype adapting to the same conditions. These phenomena therefore tell us about the stochasticity and limitations of adaptation. The limited data on convergence and parallelism in the adaptation of different genotypes conflict as to the importance of such events. If the effects of beneficial mutations are highly context dependent (i.e., if they are epistatic), different genotypes should adapt through different mutations. Epistasis for beneficial mutations has been investigated but mainly through measurement of interactions between individually beneficial mutations for the same genotype. We examine epistasis for beneficial mutations at a broader genetic scale by measuring the fitness effects of two mutations beneficial for the ssDNA bacteriophage ID11 in eight different, related genotypes showing 0.3–3.7% nucleotide divergence from ID11. We found no evidence for sign epistasis, but the mutations tended to have much smaller or no effects on fitness in the new genotypes. We found evidence for diminishing-returns epistasis; the effects were more beneficial for lower-fitness genotypes. The patterns of epistasis were not determined by phylogenetic relationships to the original genotype. To improve our understanding of the patterns of epistasis, we fit the data to a model in which each mutation had a constant, nonepistatic phenotypic effect across genotypes and the phenotype-fitness map had a single optimum. This model fit the data well, suggesting that epistasis for these mutations was due to nonlinearity in the phenotype-fitness mapping and that the likelihood of parallel evolution depends more on phenotype than on genotype.

## Introduction

Parallel molecular evolution, the fixation of the same mutations in independently evolving populations, provides some of the most convincing evidence that particular substitutions are adaptive and has been surprisingly common in experimental evolution studies, often accounting for around half of the observed substitutions among replicate lineages [1]–[4]. Theoretical work has demonstrated that the probability of parallel evolution can be surprisingly high but varies inversely with the number of possible beneficial mutations [5]. As a result, when adaptations of populations of the same ancestral genotype are compared, parallel evolution provides valuable insight into the limits of adaptation and the number of accessible beneficial mutations. However, unless order effects are examined (see, e.g., Wichman et al. [1] and Bollback and Huelsenbeck [6]), comparisons among adapting populations do not provide information on the context dependence of beneficial mutations, i.e., epistasis. Studies looking at convergent [2] or parallel [4], [6] evolution among related but different ancestral genotypes provide some information on epistasis because parallel or convergent changes in that context indicate that mutations retain a beneficial effect across different genotypes, but this pattern can be obscured by the stochasticity in the evolutionary process. If beneficial mutations tend to retain their effects in different genomes, our ability to predict molecular mechanisms of adaptation for a target organism would be significantly increased by the use of data from related strains or species.

Whether a beneficial mutation retains its effect in new genotypes depends on the presence, pattern, and strength of epistatic interactions between the mutation and the other mutations present in the new genome relative to the original ancestor. The majority of studies examining epistasis for mutations have focused on deleterious mutations, but studies of beneficial mutations have recently become common. These studies have measured interactions either between pairs of mutations that were individually beneficial [7]–[9] or between combinations of mutations identified over the course of an adaptive walk [10]–[14]. The former studies address whether different mutations beneficial for a single step in adaptation could participate in later steps, whereas the latter address the number of pathways accessible to natural selection between a defined ancestral genotype and its adapted descendent. In both cases, all of the mutations considered were identified because of their large beneficial effects in a particular selective environment, and the presence of epistasis has been universal. Antagonistic epistasis, under which the benefits of mutations are less than expected, seems to dominate for beneficial mutations [9], [13], and many beneficial mutations show sign epistasis, becoming deleterious in the context of others [7]–[9]. In all of these cases, however, the genetic backgrounds compared for a particular mutation differed by one to a few mutations. Furthermore, mutations that were selected to function together or to affect the same underlying phenoptypes might be more likely than random mutations to show strong epistatic interactions.

Most divergence between species or genotypes is due to drift and natural selection in different selective environments for different phenotypic properties and involves many individual mutations. We sought to determine whether this more generalized divergence can impinge epistatically on particular beneficial mutations. To determine whether beneficial mutations retain their benefits across diverse genetic backgrounds, we selected two beneficial mutations from the set of nine previously identified for the ssDNA bacteriophage ID11 [3], [9], [15]. The original mutations affected two different genes. We selected the two mutations of largest effect, which included one from each gene. We retain the nomenclature of previous work [9], in which the nine mutations were labeled A–I, and refer to our two mutations as B and F. The B mutation causes a V to L amino-acid change at position 20 in the J protein, which is the DNA-binding protein. The F mutations causes a P to S change at position 355 of the F protein, which is the major coat protein. Both mutations increase growth rate (i.e., fitness) in liquid culture at 37°C on *Escherichia coli* strain C. These two mutations were added, by means of site-directed mutagenesis [9], to eight new genotypes from a set of previously described natural phage isolates [16], and their fitness effects were measured by means of growth-rate assays [17]. The new genotypes differed from ID11 at 0.3–3.7% of their nucleotide sites throughout the whole genome. With genome sizes of 5529–5577 nucleotides, these divergences correspond to genomes with 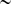
15–200 nucleotide differences from ID11.

## Results and Discussion

### Fitness Effects of Beneficial Mutations in New Genetic Backgrounds

**Figure 1:**
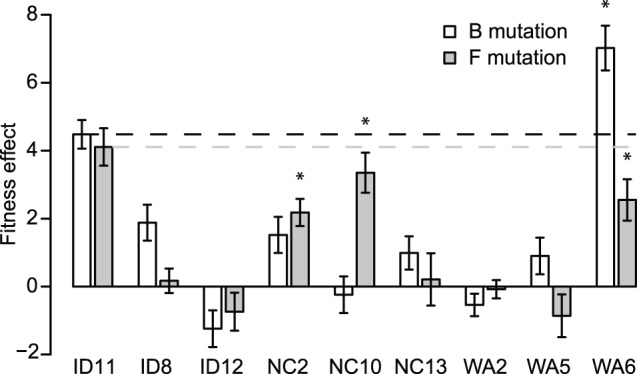
Fitness effects across genotypes of the ssDNA bacteriophage growing on *Escherichia coli*. Error bars give standard errors. We cannot reject a mean effect of zero for addition of either mutation to each of the eight new genetic backgrounds. Bars with “*” above them indicate effects that are significantly different from zero after a Bonferroni correction. The deviations from the dashed lines correspond to the values of 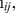 which measure the deviations from additivity.

The mean effects and standard errors of the B and F mutations in the new genotypes were 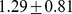 and 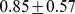 population doublings per hour, respectively. In both cases, we could not reject a mean effect of zero (B: 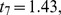 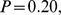 and F: 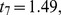 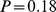). Despite the large, beneficial effects for ID11 (4.48 and 4.11 doublings per hour for B and F, corresponding to selection coefficients of 0.32 and 0.29, respectively), these two mutations tended, on average, to have little or no effect on fitness in new genotypes (Figure 1 and Table 1), but exceptions should be noted. Using Welch two-sample 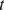-tests and a Bonferroni correction for 16 tests, we found that none of the mutations was significantly deleterious at the 5% significance level, but four were significantly beneficial: B in WA6 and F in NC2, NC10, and WA6. These results are consistent with the presence of magnitude epistasis but fail to provide evidence for sign epistasis [18] for beneficial mutations.

**Table 1:** Effects of beneficial mutations in new genotypes.

Genotype	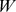	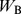	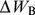	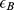	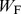	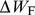	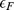	% div.
ID11	14.18±0.20	18.67±0.37	4.48	–	18.29±0.51	4.11	–	–
ID8	13.85±0.28	15.73±0.45	1.88	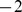 .60	14.01±0.23	0.17	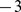 .94	3.4
ID12	19.83±0.39	18.59±0.37	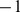 .24	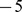 .72	19.09±0.39	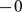 .74	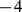 .85	3.4
NC2	13.95±0.36	15.47±0.39	1.52	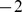 .96	16.13±0.18	2.18	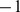 .93	3.7
NC10	6.74±0.29	6.49±0.46	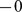 .24	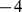 .72	10.09±0.51	3.35	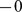 .76	3.6
NC13	13.97±0.34	14.96±0.35	0.99	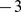 .49	14.18±0.68	0.21	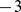 .90	2.1
WA2	12.58±0.10	12.04±0.31	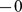 .54	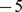 .02	12.50±0.25	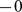 .08	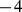 .19	3.2
WA5	17.72±0.48	18.63±0.24	0.90	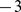 .58	16.86±0.41	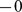 .86	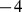 .97	0.3
WA6	6.81±0.47	13.83±0.47	7.02	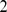 .54	9.36±0.38	2.55	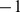 .56	3.7

Fitness values are given in units of doublings per hour plus or minus their standard errors.

In contrast to our results, Sanjuán et al. [7] and Rokyta et al. [9] measured the effects of pairs of beneficial mutations and found sign epistasis to be common. In fact, in both studies, some pairs of mutations showed decompensatory epistasis such that double mutants were less fit than either of their constituent single mutants. Sign epistasis is also common in studies decomposing adaptive walks by constructing all of the possible intermediate genotypes between the ancestor and evolved strains [10]–[14]. In contrast, parallel substitutions in different genotypes adapting to the same selective conditions can be common [2], [4] (although exceptions exist [6]), a situation that explicitly requires a lack of sign epistasis in agreement with our results. Sign epistasis may be most prominent in adapting populations in which the mutations involved have large beneficial effects potentially affecting the same or similar phenotypes or are compensatory for antagonistic pleiotropic effects of beneficial mutations. Genotypes that have diverged more extensively through drift or adaptation to different environments may be less likely to harbor strongly epistatic mutations for beneficial mutations under new selective conditions that potentially affect previously less-significant phenotypes.

### Epistasis for Beneficial Mutations

Even in the absence of sign epistasis, the nature of epistatic interactions can be further characterized by measurement of deviations from additivity for mutational effects. Because our fitness measure is a growth rate, if epistasis is not present, the two mutations should have the same additive effects across the eight new genotypes as for ID11. We can measure a deviation from additivity in a method similar to that of Rokyta et al. [9] by 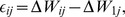 where 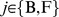 and 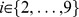 represents the eight new genotypes (Table 1). The wild-type (ID11) genotype is represented by subscript 1. Note that we deviate from the typical definition of 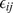 in that 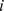 refers to a genetic background rather than an individual mutation. We are therefore measuring epistasis between one mutation (B or F) and all of the mutations in the new genotype relative to the original ancestor. Although any of our genotypes could conceivably serve as the reference genotype in the calculations of 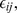 our *a priori* knowledge that the mutations have large benefits for ID11 makes this genotype the only meaningful choice. We want to know whether or to what extent the benefits conferred on the ID11 genotype by these mutations are transferred to new genotypes. Because we are dealing with beneficial mutations, 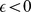 would indicate negative or antagonistic epistasis. Synergistic epistasis would give 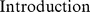 Figure 1 clearly shows a predominance of antagonistic epistasis because only a single mutant (WA6 with F) has a mean effect larger than the corresponding effect in the wild type, and all remaining cases have mean effects lower than that for the wild type. For the B mutation, 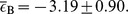 For the F mutation, 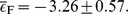 In both cases, the average deviation differs by more than three standard errors from zero. Epistasis is therefore antagonistic, a pattern that appears to be consistent for beneficial mutations [9], [13], [14]. Our results suggest more generality for this pattern than previous studies which only considered interactions between a small number of beneficial mutations. We show that, when strongly beneficial mutations are considered, this pattern also holds for the interactions between one beneficial mutation and the many mutations separating naturally diverged genotypes.

Note that the values of 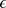 would obviously change if measured with respect to different reference genotypes (Table 2), but we only know that the B and F mutations are large-effect beneficial mutations for ID11. The other ancestral genotypes might have yielded entirely different sets of beneficial mutations under the same selective pressures or, even if the B and F mutations were beneficial, their effects might have been small relative to other accessible beneficial mutations. Nonetheless, for the four cases for which mutations were significantly beneficial in backgrounds other than ID11 (B in WA6 and F in NC2, NC10, and WA6), we found that 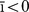 for the mutation assuming the corresponding ancestor as reference (Table 2).

**Table 2:** Deviations from additivity assuming different reference genotypes.

Genotype	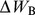	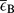	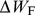	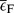
ID11	4.48	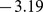 ±0.90	4.11	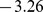 ±0.57
ID8	1.88	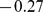 ±0.99	0.17	1.17±0.69
ID12	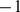 .24	3.24±0.90	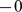 .74	2.19±0.64
NC2	1.52	0.14±0.99	2.18	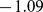 ±0.69
NC10	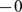 .24	2.12±0.95	3.35	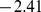 ±0.63
NC13	0.99	0.73±0.98	0.21	1.12±0.69
WA2	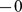 .54	2.45±0.94	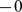 .08	1.45±0.68
WA5	0.90	0.83±0.98	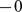 .86	2.33±0.64
WA6	7.02	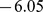 ±0.63	2.55	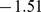 ±0.68

Fitness effects are given in units of doublings per hour, and deviations from additivity are given plus or minus their standard errors.

**Figure 2:**
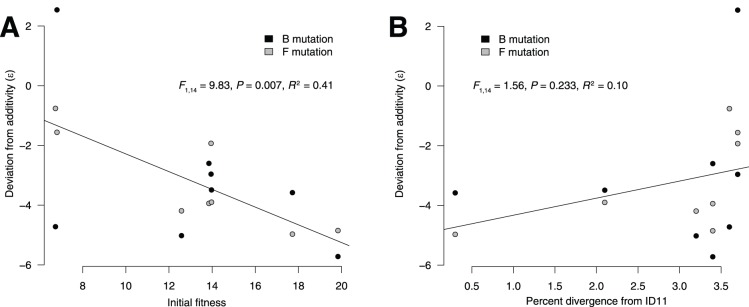
The deviation from additivity was negatively correlated with ancestral fitness. (A) Genotypes with lower initial fitnesses tended to gain more benefit from the B and F mutations than those with higher initial fitnesses, consistent with diminishing-returns epistasis. This relationship was statistically significant, and the regression line is shown. (B) We found no significant relationship between the genetic distance from ID11 and the deviation from additivity. The regression line is shown.

A number of factors could contribute to the deviations from additivity for the B and F mutations. Most obviously, we expect that the effects of the mutations should be more similar to the effects in ID11 for genotypes that are more closely related to ID11 [6]. The location of isolation, being a proxy for similarity of historical selective pressures, might have led to distinct phenotypic properties among the isolates that could contribute. Bull et al. [19] noted that, for the related phage 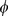
X174, the magnitude of the beneficial effect of a mutation could be negatively correlated with the fitness of the genotype into which it is added. We might therefore expect the mutations to have larger effects (and therefore be closer to additive) in genotypes with lower initial fitnesses. Finally, idiosyncratic properties of the mutations themselves could contribute, if, for example, one mutation exhibited stronger epistatic interactions than the other. We tested for these effects using an ANCOVA analysis in R [20]. We used 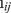 as our response variable and included mutation identity (B or F) and isolation location (ID, NC, or WA) as categorical explanatory variables. Percent genetic divergence from ID11 (Table 1) and the initial (i.e., ancestral) fitness of the genotypes were included as continuous explanatory variables. In the full model, the only significant explanatory variable was initial fitness (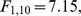 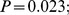 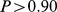 for all other variables); the mininum adequate model, including only initial fitness, gives 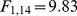 and 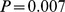 (Figure 2). With 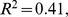 however, the model provided a poor explanation for our data. The estimated slope was negative, indicating that genotypes with lower initial fitnesses received closer-to-additive effects. The data were slightly heteroscedastic, primarily on the basis of the WA6 genotype with the B mutation. Eliminating this genotype from the analysis had little effect on the result (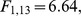 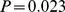). We found, therefore, that epistasis tends to be more antagonistic for genotypes with higher ancestral fitnesses. This pattern corresponds to diminishing-returns epistasis, which has been observed for beneficial mutations for the related bacteriphage 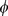
X174 [21] and in bacteria [13], [14], [22], [23].

### Epistasis and the Phenotype-fitness Map

**Figure 3:**
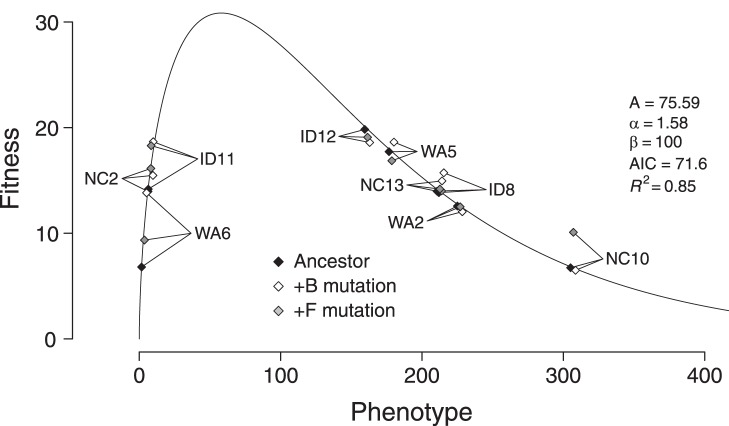
The estimated phenotype-fitness map under the gamma model. Addition of the B mutation moves each ancestral genotype the same distance and direction along the horizontal axis, as does the F mutation. Interestingly, the two genotypes most distantly related to ID11 (the original ancestor for identifying the mutations), NC2 and WA6, are its closest neighbors in phenotypic space.

By looking just at fitness effects, we were unable to explain most of the variation in our data. In previous work characterizing epistasis between pairs of beneficial mutations [9], a model with additive phenotypic effects fit the data well. Epistasis was incorporated at the fitness level by means of a nonlinear phenotype-fitness map. The model was based on the observation that biochemical effects of mutations are often additive [10], [24] and the assumption of an intermediate phenotypic optimum (i.e., stabilizing selection). Each mutation was assumed to affect a single phenotype, and its phenotypic effect was the same regardless of the background genotype. The phenotype of the mutant was then mapped to fitness by means of a gamma curve to allow for asymmetry in the phenotype-fitness map. To determine whether a similar model could explain our data, we imputed phenotypic values from fitnesses and estimated a phenotype-fitness curve. We assumed a one-dimensional phenotypic space and that the phenotype-fitness relationship followed a gamma function
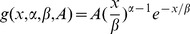
with shape parameter 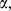 scale parameter 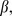 and height parameter 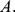 The phenotype is denoted by 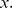 Note that the height parameter 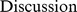 does not equal the maximum height of the curve but is instead a scaling factor. Also note that the scale of the phenotypes is arbitrary; to avoid the infinite number of possible scalings for the 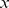-axis, we set 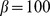 throughout our analyses. We assumed that each of nine ancestral genotypes had a phenotype 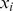 for 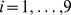 and that each mutation had an additive phenotypic effect (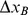 and 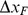) that was constant in direction and magnitude across all backgrounds. As an example, the fitness of the B mutation in genotype 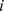 is given by 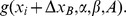 We estimated the parameters and imputed phenotypes and phenotypic effects by nonlinear least-squares regression (Figure 3). See the Supporting Information online for details on the optimization procedure and its limitations.

**Figure 4:**
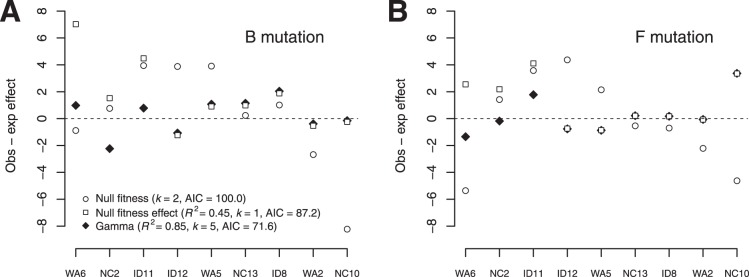
A comparison of model fit for the gamma model and the two null models for the B mutation (A) and the F mutation (B). Fitness effects were measured in units of doublings per hour. For the null fitness model, we assumed that the fitnesses of the mutants were normally distributed and estimated the mean and variance. For the null fitness effect model, we assumed that the effects of mutations were normally distributed with mean zero and estimated the variance. The numbers of parameters in the models are given by 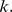 Note that the null fitness model was used as the basis of our 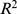 calculations and therefore has no 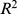 value associated with it.

To assess model fit, we conducted two different tests (Figure 4). First, we computed a coefficient of determination 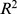 by assuming that the uninformative null model was that the fitness of every mutant genotype was an independent draw from the same normal distribution. This procedure allowed us to calculate the total sum of squares. We found that, relative to this model, the gamma model explained 85% of the variation in the data, indicating good fit for the model that assumes phenotypic effects are independent of genetic background. For the second test, we determined whether we could reject an alternative model that assumed nothing about the underlying phenotypic effects of the two mutations. For this model, the fitness effects of the mutations in all backgrounds were assumed to be normally distributed with mean zero and variance estimated from the data. Relative to this null model, the gamma model explained 73% of the variation in fitness effects. Using an 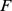 test, we could reject this model (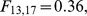 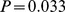). We could also easily reject our two null models with Akaike information criteria (AIC) scores. For the gamma model, we got an AIC of 71.6. For the model used for calculating 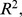 the AIC was 100.0, and the AIC was 87.2 for the second null model. Again, we found support for a model in which phenotypic effects are additive across genotypes and epistasis arises from the nonlinearity of the phenotype-fitness map.

Our goal with the model was to explain the fitnesses and fitness effects of the mutants by imposing structure on the underlying, unobserved phenotypes. To further confirm that the model was discerning a real pattern in the data, we performed a randomization test on the fitness effects of the mutations. We generated 500 data sets for which the 18 observed fitness effects were assigned at random to the nine ancestral genotypes. We then conducted a likelihood-ratio test by calculating the log-likelihood of the data under both the gamma model and a null model corresponding to the randomization procedure (mutational effects are normally distributed with mean 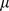 and variance 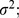 see Supporting Information), taking the difference, and determining how often a value as large as the difference observed in the real data was observed among the bootstrap data sets. We found that in none of the 500 data sets did the bootstrap difference meet or exceed the observed difference (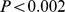). The model therefore fit the data not just because it predicted the approximate fitnesses of each set consisting of an ancestor and its two mutants, but also because it described the patterns of epistasis within each set.

Because our set of genotypes, consisting of two mutations introduced into nine ancestors, is of modest size, the generality of our results is difficult to assess. We were able to reject our null models, which demonstrates that statistical power is not an issue, but whether the inclusion of additional mutations would significantly alter the overall results is uncertain. We selected the two largest-effect mutations of the original set of nine because these mutations were the most likely to show strong epistatic effects. These mutations were therefore also most likely to have the strongest influence on the fit of the model. Despite these mutations affecting different genes, their epistatic patterns were similar enough that mutation identity was not found to be a significant factor in our ANCOVA, which suggests that the patterns for additional mutations would be similar.

**Figure 5:**
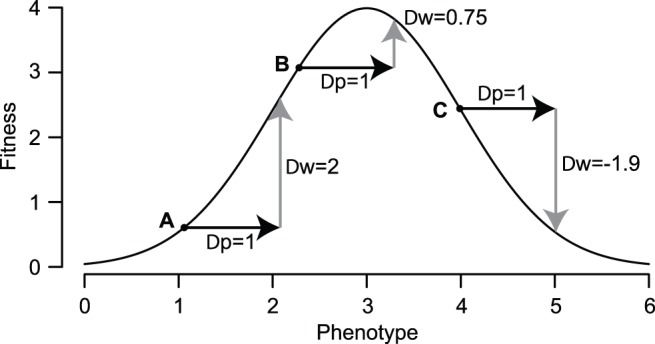
Nonlinearity in the phenotype-fitness relationship can produce commonly observed epistatic patterns. Three hypothetical genotypes (A, B, and C) with different phenotypes are all given the same mutation with the same phenotypic effect (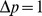). If the mutation were moved from background A to background B, we would see antagonistic epistasis. Moving it from B to A would give synergistic epistasis, and moving it from A to C would give sign epistasis.

All major types of epistasis can emerge naturally from the additive phenotypic model because the relationship between phenotype and fitness is nonlinear (Figure 5). Antagonistic epistasis, under which the effects of mutations are less than additive, can arise when a mutational vector is moved from its original genotype to a new genotype in a region with a more concave phenotype-fitness relationship. Likewise, synergistic epistasis, in which the effects of mutations are more than additive, can arise when a mutation is moved to a region with a more convex phenotype-fitness relationship. Sign epistasis [18], e.g., the case in which a beneficial mutation becomes deleterious in a new background, can arise when a mutation overshoots the optimum in one genotype but not another (Figure 5). Understanding epistasis is critical for understanding, for example, the rate of adaptation [13], the evolution of sexual reproduction [25], [26], and reproductive isolation between incipient species [27], [28]. Under this model, epistasis is not a mysterious intrinsic property of particular genotypes but a reflection of the curvature of the phenotype-fitness map [29].

The most obvious limitation of our analysis is the restriction to a single phenotypic dimension. Had we rejected the model, this restriction might have been significant, because assuming more phenotypes would have improved the fit of the model. Though the two mutations affect different genes, they can reasonably be assumed to affect the same single phenotype. Despite being in different genes, the mutations lie in the same region of the capsid structure; the 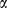-carbons for the positions of the B and F mutations are separated by only 19.2 Å in the structure of the related phage 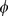
X174 [30], [31]. Perhaps more troublesome is the assumption that the ancestral genotypes differ primarily in this one phenotype. Rokyta et al. [4] adapted eight different microvirid genotypes, including two of those from the present study, to the same culture conditions used for the study reported here. They found that adaptation took on average three substitutions and that parallel evolution was common even across different genotypes. These two observations suggest that the phages are not maladapted in many ways and that the same traits are responding to selection in the different genotypes. In effect, our approach can be viewed as being similar to a principal-components analysis in which we try to identify a single axis in phenotypic space that best explains our data.

The model described above is effectively a one-dimensional version of Fisher’s geometric model [32]. Fisher’s model has defined how evolutionary biologists think about the genetics of adaptation since its introduction more than 80 years ago [32]. It led directly, for example, to the generally accepted expectation that adaptation should occur primarily through small-effect mutations, an expectation that has not always been met empirically [15], [19], [33] and that has since been shown to be an overly simplistic view of the implications of the model [34]–[36]. The assumption is that, to be fit, an organism must match its environment in some number of phenotypes. Each of these phenotypes is treated as an orthogonal axis in a continuous metric space. The evolving genotype then moves toward the optimum by means of selection acting on mutational vectors that can point in any direction in the space, affecting one or more phenotypes. This framework has been used to study, for example, the probability that a mutation is beneficial [32], the evolution of sex [37], development [38], dominance [39], the cost of complexity [40], and epistasis [29]. Epistasis is a phenomenon resulting from how phenotypes are translated into fitness [29]. Phenotypic effects are additive under Fisher’s model, but fitness effects may not be. If correct, Fisher’s model provides a simple explanation for patterns of epistasis and implies that most of the interesting biological phenomena might arise in the relationship between phenotypes and fitness, rather than genotype and phenotype. Importantly, the genotype-phenotype relationship, usually involving a complex developmental pathway, is much more difficult to characterize than the phenotype-fitness relationship. When we fit a model in which mutations are nonepistatic phenotypic vectors, our data are consistent with Fisher’s model.

In previous work with these mutations [9], phenotypic effects of individual beneficial mutations were determined to be additive when the mutations were added as pairs to the same genotype by means of a modeling framework similar to that of the present study. These results only address epistasis for mutations between genotypes differing by at most one mutation and therefore only establish applicability of the model to small, local regions of genotypic space. In contrast, the genotypes in this study differ by from tens to hundreds of mutations with unknown effects and therefore provide a broader test of the model, dramatically increasing the generality of the result. Previous results also provided support for Fisher’s model [29], [41] but tested downstream predictions of the model, which rely on assumptions about the phenotype-fitness map, distribution of mutations, and distance from the optimum. We tested the underlying assumption upon which these others are built: that mutations behave like vectors in phenotypic space.

Our results, though limited in scope to two mutations in nine genetic backgrounds, could have important implications for understanding epistasis and parallel evolution. Whether different genotypes or species adapt through the same mutations to similar selective pressures could depend more on phenotypic properties than on simple genetic distance. In other words, parallel evolution might be more likely between phenotypically similar organisms; propinquity in genotypic space might be important only insofar as it predicts the relationship in phenotypic space. This pattern may serve as a mechanism for historical effects to impinge on the predictability of adaptive evolution and suggests the testable hypothesis that species with more similar past selective pressures are more likely to adapt by similar mechanisms even if they are not particularly closely related. For epistasis, our results show that the complex patterns for epistatic interactions are likely to be unpredictable from a knowledge of genotype alone and will probably change dramatically across environments as the phenotype-fitness map shifts. Rather than focusing on the identification of the most abundant class of epistatic interaction (e.g., antagonistic or synergistic), a more informative focus might be the common forms of phenotype-fitness maps.

## Materials and Methods

### Constructing the Mutants and Fitness Assays

The ancestral bacteriophage genotypes used in this study were unadapted, natural isolates from wastewater facilities or barnyards [16]. Our NC2 ancestor had a silent nucleotide change of C to T at position 961 relative to the published sequence. The nucleotide change was in codon 301 for the A gene and 88 for the A* gene. NC13 had a nonsynonymous change at position 3122 of T to C, resulting in an amino-acid change of S to P in position 174 of gene F, the major coat protein gene. WA2 had two nucleotide changes: a silent G to T transversion at nucleotide position 433 (amino-acid position 125 in gene A) and a nonsynonymous C to A transversion at position 1465. This transversion resulted in an amino-acid change for genes A and A* (amino-acid positions 469 and 256, respectively) from N to K and was also silent in gene B at amino-acid position 64. The differences from the published sequences either represent sequencing errors in the originals or mutations acquired by chance in the choice of isolates for the present study. Because the differences are present in both the ancestors and the constructed mutants, they should have no effect on our results.

The isolation and characterization of the two beneficial mutations from the microvirid bacteriophage ID11 have been previously described [3], [15]. The B mutation is a G to T transversion at nucleotide position 2534, resulting in an amino-acid change of V to L at position 20 of the J protein. The F mutation is a C to T transition at nucleotide position 3665, resulting in an amino-acid change from P to S at position 355 of the F protein. The sites of these mutations in the eight other genotypes were determined on the basis of homology; the actual nucleotide position varied with genome length.

The mutants were created by PCR-mediated site-directed mutagenesis [9], [42], [43]. The circular ssDNA genomes were PCR amplified in two halves. Amplification of one half was conducted with a forward primer containing the mutation to be inserted, and for the other half, a reverse primer with the mutation was used. The other primers were selected to ensure overlap between the halves. The two halves were combined in a PCR without primers. The full genomes were electroporated into *E. coli* strain C, and we confirmed each mutant genotype by full-genome sequencing.

Fitness was measured as the log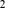 increase in the size of the phage population per hour of growth on *E. coli* C at 37

C as described previously [17], [43]. The assays were conducted in an orbital water bath shaking at 200 rpm. Hosts were allowed to grow for one hour in liquid medium (10 g/l NaCl, 10 g/l tryptone, 5 g/l yeast extract, and 2 mM CaCl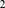) before the addition of phage. After 40 minutes of phage replication, a 1 ml sample was extracted from the flask, and chloroform was added to terminate the infection. Phage titers before and after the growth period were measured by standard plating assays. The fitness of each genotype was measured at least five times.

### The Phenotype-fitness Map

To determine whether phenotypes were additive, we assumed that a gamma function described the relationship between phenotype and fitness for all 27 genotypes
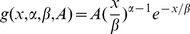
where 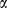 is the shape parameter, 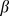 is the scale parameter, 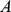 is the height parameter, and 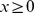 is the phenotype. We used the gamma function (not distribution) to allow for a flexible, potentially asymmetric map. Each of the nine ancestral genotypes was assumed to have a unique and independent phenotype. The two mutations, B and F, were assumed to have additive phenotypic effects on all backgrounds. The phenotype for a B mutant on background 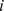 is 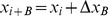 for all 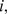 and an F mutant phenotype is similarly 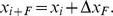 The measured fitnesses of the nine genetic backgrounds were then given under the model by 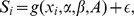 the fitnesses of the B mutants were 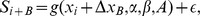 and those of the F mutants were 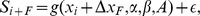 where 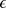 is normally distributed with mean zero and variance 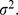 Note that under the gamma model, any rescaling of phenotypes 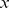 can be compensated for by a corresponding change in the scale parameter 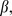 yielding an infinite number of curves with exactly the same fit to the data and shape. Because our phenotype values are arbitrary, we set 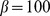 to simplify the optimization problem.

### Parameter Optimization

We found the optimal set of gamma parameters (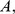 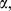 and 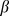) and imputed phenotypes (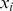 for all backgrounds 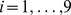) and phenotypic effects (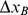 and 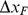) by minimizing the sum of squared deviations from the mean observed fitness values given by
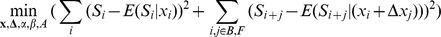
where 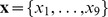 is the set of ancestral phenotypes, and 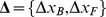 are the phenotypic effects of the two mutations. The phenotypes and phenotypic effects were treated as missing data. The values denoted by 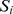 and 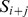 are the observed fitnesses, and the expected values of these are given by the model. An iterative optimization procedure was initiated by defining gamma parameters. Because the curve is peaked, it has two roots for each fitness value. We located the two roots for each background given its fitness; 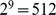 combinations of these roots (512 possible phenotype sets) are possible for the observed data. For each of these, we found the values for 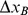 and 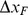 that minimized the sum of squares by calculating, for each of the 512 possible phenotype sets, the sum of squares for a range of values of 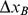 and 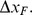 We selected the least-squares values for 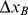 and 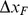 and summed them to get the least squares for each phenotype set and repeated over all 512 sets. We selected the phenotype combination and values of 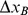 and 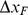 that had the overall smallest sum of squares. By a similar strategy, we found the joint set of gamma parameters that minimizes sum of squares given the set of phenotypes and values for 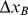 and 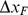 obtained above. These steps were repeated until convergence. When we ran this optimization algorithm on the real data set beginning with different initial gamma values, it produced different estimates indicating that the likelihood surface is multimodal. Consequently, we ran the algorithm from 20 different initiation points in parameter space. The best likelihood score over the 20 runs defined the estimates (see Supporting Information for details). Note that, for our purposes, we only need a good fit, not necessarily the best fit, especially given that we were able to reject our null models with the optimum we found (see below). Note that we have 27 observations (a wild type, B mutant, and F mutant for each of nine genotypes). We estimated nine phenotypes, two phenotypic effects, two gamma parameters, and the error variance, leaving 13 degrees of freedom.

### Assessing Model Fit

We assessed model fit by calculating a coefficient of determination 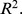 To calculate the total sum of squares, we constructed a model that assumes that every mutant genotype is a sample from the same normal distribution with estimated mean and variance. This procedure is equivalent to assuming that the fitness of the mutants is independent of the ancestral background. For this model, we estimated a mean and a variance and estimated the ancestors’ fitnesses directly, leaving 16 degrees of freedom. We also conducted an 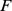 test against a model where mutations confer fitness effects that are normally distributed with mean zero and some variance, which are added to the appropriate ancestral fitnesses. This procedure is equivalent to assuming that phenotypic effects have no underlying structure (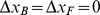) and that mutations are equally likely to be beneficial or deleterious. For this model, we only estimated a variance, leaving 17 degrees of freedom. We then compared both models to the gamma model with Akaike information criterion (AIC) values. Finally, to determine whether the explanatory power of the gamma model was merely the result of fitting the clusters of fitness values (i.e., the fitness of each background and its two correlated mutant fitnesses) or was the result of also explaining the patterns of epistasis within clusters, we randomly assigned the 18 observed fitness effects among the nine backgrounds (without replacement), thereby maintaining the fitness clusters but eliminating any epistatic patterns within them, and conducted a likelihood-ratio test. For each of 500 randomized data sets, we calculated the log-likelihood under the gamma model and under a null model corresponding to the randomization process (fitness effects were normally distributed with mean 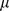 and variance 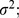 see Supporting Information for details), took the difference, and determined how often this difference in log-likelhoods for the real data was as large or larger than the difference for the randomized data. Note that in all of the model comparisons described above, parameter estimation under the null models required simple estimation of means and variances of fitnesses or fitness effects, but estimation under the alternative model required complex optimization that was not guaranteed to find the global optimum. The comparisons were therefore biased against the alternative model, making them conservative.

## Supporting Information

Supporting Information S1 **Details of the optimization procedure and a discussion of its limitations.**
(PDF)
