# Supplementary material for: The Consistency of Beneficial Fitness Effects of Mutations across Diverse Genetic Backgrounds
Source: PLoS One. 2012 Aug 24;7(8):e43864. doi: 10.1371/journal.pone.0043864 (PMC3427303; doi:10.1371/journal.pone.0043864)
Supplement: Supporting Information S1 — Details of the optimization procedure and a discussion of its limitations. (PDF) [file pone.0043864.s001.pdf]

Supporting Information—Optimization and Randomization Test

For our phenotype-fitness model, we assumed that a gamma function

$$g(x, \alpha, \beta, A) = A \left(\frac{x}{\beta} \right)^{\alpha - 1} e^{-x/\beta}$$

describes the phenotype-fitness relationship and that mutations have additive phenotypic effects. We estimated the gamma parameters, the background phenotypes, and the phenotypic effects of the two mutations. Note that, under the gamma model, any rescaling of phenotypes x can be compensated for by a corresponding change in the scale parameter β, yielding an infinite number of curves with exactly the same shape and fit to the data. Because our phenotype values were arbitrary, we set $\beta = 100$ to simplify the optimization problem. Our estimation procedure involved maximizing the likelihood in an iterative fashion. We began with a set of fixed gamma parameters that defined a curve and then estimated the phenotypes of the backgrounds and the phenotypic effects of the mutations that maximize the likelihood by means of least squares. Next, we fixed these phenotypes and phenotypic effects and found the gamma curve that maximized the likelihood. We then iterated until convergence. This procedure breaks the dimensionality of the problem into two parts and climbs the likelihood surface by moving in a subset of dimensions each time. The shortcoming of this procedure is that we are only assured of climbing uphill from the initial set of parameters; if the likelihood surface has more than one peak, the search may not find the global optimum. We investigated whether this issue was a problem for our data by initiating the search from a number of different starting points. We found that the algorithm does not always converge on the same estimate from different starting points, but that this behavior has no effect on our conclusions. Note that in all of the model comparisons we make, parameter estimation under the null models requires simple estimation of means and variances of fitnesses or fitness effects, but estimation under the alternative gamma model requires complex optimization that is not guaranteed to find the global optimum. The comparisons are therefore biased against the alternative model, making them conservative.

We explored the issue of local optima by initiating the optimization algorithm at 20 different gamma curves with a diversity of shapes, from highly asymmetrical to nearly symmetrical, and heights, from peaks just above the largest observed fitness to extremely high peaks (Figure S1). Note again that the x axis (phenotype) has an arbitrary scale defined by $\beta = 100$. After running the 20 optimizations to convergence, we identified the replicate with the best log-likelihood score as our estimate. We also considered other runs within two log-likelihoods of the best to be good explanations of the data. For the real data, Figure S2 shows the five curves falling within 2 log-likelihoods of the best. Qualitatively, the six curves are similar, having steep slopes on the left and more gradual slopes to the right. Figure S3 provides the details of each of these six curves and the phenotype data associated with them. Note that backgrounds ID8 and WA2 flip between the right and left sides of the peak. These results demonstrate the uncertainty surrounding some parameter estimates but also show that this uncertainty has little effect on the fit of the model. A range of asymmetrical curves with positive skew is consistent with our data and, although the qualitative order of background phenotypes is generally quite similar, some changes are present.

Our focus is not on the parameter estimates themselves but rather on determining whether the gamma model and other additive-phenotype models are productive ways to study and elucidate epistasis and adaptive evolution. To that end, we must be sure that the gamma model has real explanatory power and is not fitting noise or simply overparameterized. We tested this possibility in several ways, including an F-test, the AIC, and a randomization procedure. Because the randomization procedure is quite similar to the way in which the real data were analyzed, we provide a detailed account of the procedure below.

Our approach was to use a likelihood-ratio test, which involves the ratio of the likelihood under the full model and a null model. In order to determine whether the gamma model explains a significant amount

of the variation in the data, we must therefore specify a null model. Several null models are possible; we considered three closely related ones. We could assume that, across all backgrounds, the two mutations (a) each have their own mean fitness effect (2-effect null), (b) have a single mean fitness effect (1-effect null), or (c) have no mean effect on fitness (no-effect null). For all three, we assumed that deviations from expectation followed a normal distribution with a variance that must be estimated from the residuals. The models were compared on the basis of AIC. On the basis of the results ($\mathrm{AIC}_{\text{2-effects null}} = 84.5$, $\mathrm{AIC}_{\text{1-effect null}} = 82.6$, $\mathrm{AIC}_{\text{no-effect null}} = 87.2$), we selected the 1-effect null for the randomization test.

We calculated the log-likelihood under both the 1-effect null model and the gamma model. We defined the test statistic as twice the difference in log-likelihood (equivalent to taking the ratio of their likelihoods): $\Lambda = 2 \ln L_{\text{gamma}} - 2 \ln L_{\text{null}}$. Let the value of Λ calculated for the real dataset with the best parameter estimates be Λ_{obs}. We then determined how often a value as large as or larger than Λ_{obs} was observed when the null model is true. When such values are sufficiently rare, we can reject the null model. We addressed this problem by simulating the distribution of Λ under the null and determining where in the resulting distribution Λ_{obs} fell. To accomplish this task, we took the 18 observed fitness effects in the data and reassigned them to backgrounds at random 500 independent times. We thus randomized the fitness effects in the data, consistent with the null model. For each randomized data set, we fit the data to the gamma model, in exactly the same way as for the real data. We began the optimization procedure from each of 20 gamma curves and used the replicate with the best likelihood as the point estimate. We then calculated the log-likelihood under the gamma model and the null model and took twice their difference to obtain an observation of Λ_{rand}. The results from this analysis are presented in Figure S4. For the real data, $\Lambda_{\text{obs}} = 16.84$. Over the 500 replicates, the largest value of Λ_{rand} we obtained was 13.60. Therefore $P < 0.002$, strong evidence that the gamma model explains a significant amount of the variation in fitness effects.

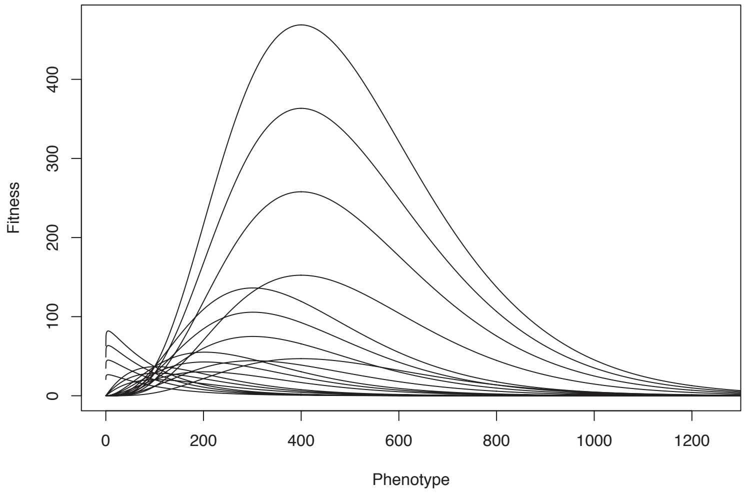

Figure S1. The 20 gamma curves from which optimization was initiated. These curves were selected to provide a representative set of gamma curves from highly asymmetrical to nearly symmetrical. We constrained all curves to have $\beta = 100$.

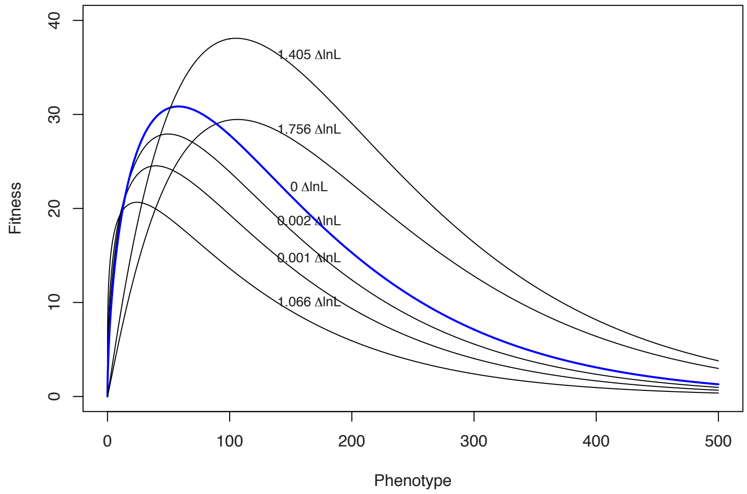

Figure S2. The maximum likelihood gamma curve and the five other curves falling within 2 log-likelihoods of the best. The curve shown in blue was the best curve found by our optimization procedure. The other curves also provide good explanations for our data.

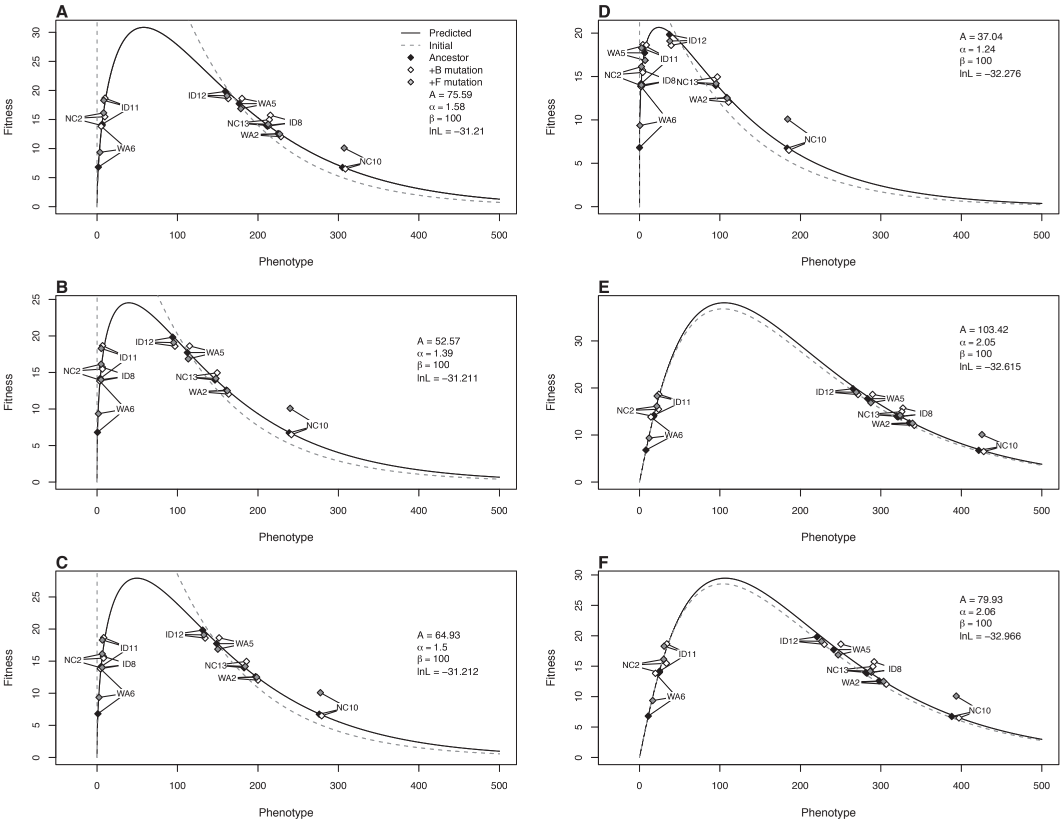

Figure S3. The six best sets of parameter values presented in descending order of log-likelihood (A–F). The dashed lines indicate the initial curve that resulted in each set of estimates.

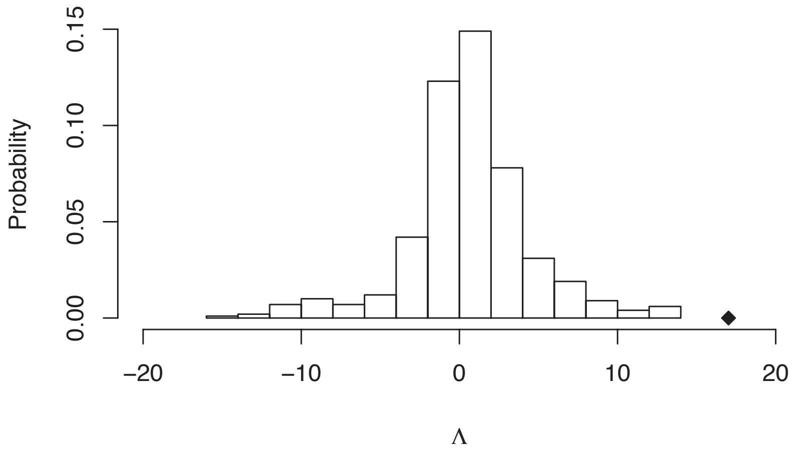

Figure S4. Simulated distribution of Λ under the null model and the location of Λ_{obs} (black diamond) relative to this distribution.
